# Economic impacts of health shocks on households in low and middle income countries: a review of the literature

**DOI:** 10.1186/1744-8603-10-21

**Published:** 2014-04-03

**Authors:** Khurshid Alam, Ajay Mahal

**Affiliations:** 1 Monash School of Public Health and Preventive Medicine, Monash University, The Alfred Centre, 99 Commercial Road, Level 5, Melbourne, VIC 3004, Australia; 2 Equity and Health Systems, International Centre for Diarrhoeal Disease Research, Bangladesh (ICDDR,B), Mohakhali, Dhaka 1212, Bangladesh

**Keywords:** Health shocks, Low- and middle-income countries, Catastrophic out-of-pocket health spending, Impoverishment, Labour supply and income loss, Non-medical consumption effect, Coping strategies

## Abstract

Poor health is a source of impoverishment among households in low -and middle- income countries (LMICs) and a subject of voluminous literature in recent years. This paper reviews recent empirical literature on measuring the economic impacts of health shocks on households. Key inclusion criteria were studies that explored household level economic outcomes (burden of out-of-pocket (OOP) health spending, labour supply responses and non-medical consumption) of health shocks and sought to correct for the likely endogeneity of health shocks, in addition to studies that measured catastrophic and impoverishment effects of ill health. The review only considered literature in the English language and excluded studies published before 2000 since these have been included in previous reviews. We identified 105 relevant articles, reports, and books. Our review confirmed the major conclusion of earlier reviews based on the pre-2000 literature - that households in LMICs bear a high but variable burden of OOP health expenditure. Households use a range of sources such as income, savings, borrowing, using loans or mortgages, and selling assets and livestock to meet OOP health spending. Health shocks also cause significant reductions in labour supply among households in LMICs, and households (particularly low-income ones) are unable to fully smooth income losses from moderate and severe health shocks. Available evidence rejects the hypothesis of full consumption insurance in the face of major health shocks. Our review suggests additional research on measuring and harmonizing indicators of health shocks and economic outcomes, measuring economic implications of non-communicable diseases for households and analyses based on longitudinal data. Policymakers need to include non-health system interventions, including access to credit and disability insurance in addition to support formal insurance programs to ameliorate the economic impacts of health shocks.

## Introduction

Health shocks, whether an event of death or disease, can cause significant adverse economic outcomes for households low- and middle-income countries (LMICs). Poor health among members can increase the risk of a household becoming destitute if there are significant out-of-pocket (OOP) healthcare expenditures incurred to obtain healthcare. Even if OOP treatment costs are avoided by not seeking care, the household to which a sick individual belongs may still forgo earnings if there are work-days lost by the sick individual or his informal caregivers. From a policy standpoint, any adverse economic outcomes of health shocks of households hinder progress on national development goals such as poverty reduction and economic growth. Excessive reliance on OOP health spending may also ration scarce healthcare services away from the less well-off to those who can afford to pay, enhancing inequalities in access to care [1]. Intergenerational equality may also be affected, if health shocks adversely influence national and household ability to contribute to child health and educational outcomes.

Analyses of the economic impacts of health shocks have been the subject of significant researcher attention in recent years, outpacing existing reviews of the economic implications of ill health, which are either limited in scope or out of date. Of the three previous reviews most relevant for this paper, Russell [2] explored the direct and indirect cost of illness related to HIV, tuberculosis and malaria; McIntyre *et al.*[3] focused on household level impacts of OOP medical spending and labour-days loss due to illness in the pre-2000 literature; and Acharya *et al*. [4] focused on protective effects of voluntary insurance from the economic implications of illness, including recent insurance interventions. However, considerable gaps remain. In particular, the post-2000 literature on the implications of illness for non-medical consumption, labour supply and informal coping mechanisms has simply not been covered in existing reviews with one exception: and that exception, Acharya *et al.*[4], only assessed the impacts of insurance programs on household OOP spending in LMICs.

The primary goal of this paper is to summarize recent evidence on the economic impacts of health shocks in LMICs based on the World Bank definition of LMICs as countries with a gross national income per capita less than US$ 12,616 in 2012. The overarching research question guiding this review was: what are the economic impacts of health shocks on households in LMICs and what factors influence the magnitude of these impacts? The specific sub-questions were: what are the impacts of health shocks on OOP health payments of the households and on measures of catastrophic spending and impoverishment? What are the impacts of health shocks on labour supply and earnings of households? What are the impacts on non-medical consumption of the households? What contextual and other factors influence the magnitude of these impacts? Our review adds to earlier reviews of the literature by bringing together a large number of recent studies, and specifically during 2000–2014, in the LMICs of Asia, Africa, Latin America and Eastern Europe on the economic impacts of health shocks at the household level. In addition, advances in the methodology of estimating the impacts of health shocks and new ways of measuring the economic burden of illness developed in recent years mean that our review adds significantly to the information base on the household economic implications of illness. Because of the existing review by Acharya *et al.*[4], we only include a brief discussion of the analyses of the implications of formal insurance programs.

## Methods

**Figure 1:**
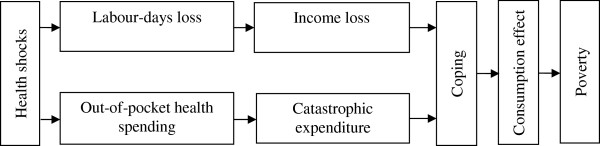
Conceptual framework of economic impacts of health shocks on households in low and middle income countries.

Figure 1 illustrates the conceptual approach that guided the current review. Specifically there are two main avenues through which impacts of health shocks are likely to be felt by households. First, households are at risk of incurring OOP health spending if they seek treatment. If the OOP spending is large, relative to say exceeding a certain threshold of a household’s income or some measure of ‘capacity to pay’, there is the possibility that it might be ‘catastrophic’ in nature (see Additional file 1 for definitions) [1,5-7]. The degree to which OOP health spending is catastrophic for the households often depends on whether social protection mechanisms exist. For instance, we would expect OOP expenses to be low if good quality subsidized public facilities are accessible to households, or if there is health insurance coverage that pays for the use of health services [8]. However, OOP can also be low if households simply forgo healthcare if they are not in position to pay for it and this may have other consequences, including poor health outcomes and loss of earnings. Second, households may face a loss of productive labour time and earnings due to illness or death of their members and associated caregiver time. If illness-affected household members or their caregivers work in the formal sector, earnings losses might be limited, but this is not common in LMICs.

To limit the consequences (OOP health spending, non-medical spending and earnings losses) of health shocks households can potentially adopt one or more of a range of strategies. These can include borrowing and sales of assets to meet expenses and earnings losses, accessing informal community-based insurance pools and formal health insurance, increase the labour force participation of young children or diversify income sources, say by better access to credit [9]. If these strategies are ineffective, households can experience significant declines in non-medical consumption, including expenditures on such items as food, education, housing and recreation. In some cases, the lowering of non-medical consumption can be so severe as to lead to the household being classified as poor (see Additional file 1) [5,10]. Households can also be impoverished in the longer run if sales of productive assets, borrowing and reduced educational investments impose a significant future financial outgo and lower household earnings.

### Search strategy

**Figure 2:**
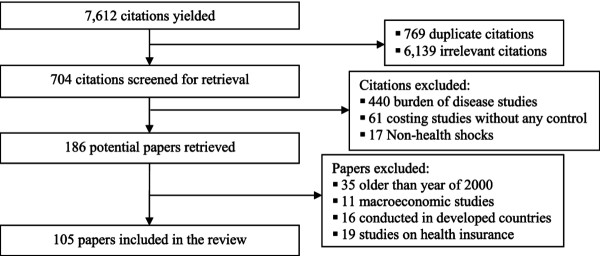
Search strategies for selection of studies exploring economic impacts of health shocks on households in low and middle income countries.

To assess the empirical evidence available on these economic impacts, we employed a comprehensive search strategy (Figure 2) using electronic databases such PubMed/Medline, EconLit, Science Direct, Social Science Citation Index, Applied Social Sciences Index and Abstracts (ASSIA), and Social Sciences Abstracts. In these search strategies we used a range of keywords relating to economic impacts of health shocks on households and coping strategies in LMICs (health shocks/illness/death, medical expenditure, OOP health payments, catastrophic expenditure, labour supply loss, income loss, non-medical consumption, poverty/impoverishment, coping strategy). Apart from generic illness or health shocks we were also interested in specific health problems such as HIV/AIDS, adult deaths, and non-communicable diseases (NCDs) considering their importance in global disease burden and we searched on these specific health conditions combining with the aforementioned same key words.

Our inclusion criteria limited analyses to household level economic outcomes due to health shocks among households living in LMICs. Macroeconomic analyses that did not assess household or individual level economic consequences, studies conducted in developed countries, and studies published before 2000 were excluded from the analysis. Our review was further limited to literature in the English language. Crucially, we limited our attention to studies that used methods to address potential biases in estimating the impacts of health shocks on household economic outcomes. The risk of bias arises because health shocks are unlikely to be truly exogenous. Indeed, two-way causality between economic outcomes and health events (classic endogeneity), unobserved characteristics of individuals that increase both the likelihood of their being more susceptible to illness and to more severe economic outcomes, or selection into specific behaviour (unobserved heterogeneity), and poor recall (measurement errors) in reports of illness and its severity could all bias estimates of the impacts of illness. There may also be simple cases of omitted variables where inclusion of additional control variables can help. For these reasons our inclusion criteria required that the methodology employed by the authors had addressed at least one (ideally more) of the estimation challenges mentioned earlier. We limited our search to studies that had relied on instrumental variable methods, fixed-effects or random-effects approaches in the context of longitudinal data, analyses where (after controlling for appropriate variables) there could be reasonable expectation that the health shock could be treated as exogenous (including quasi-experimental methods such as propensity score matching), or methods correcting for selection effects.

There was one set of exceptions, however, to the above. We also included studies that used an accounting approach to assess economic impacts - specifically studies that calculated catastrophic and impoverishing effects associated with OOP health spending on healthcare. This was done because of the overwhelming popularity of this methodology following the initial work of Xu *et al.*[6] and Doorslaer *et al.*[10], both published in the *Lancet*[6,10], despite conceptual shortcomings [11].

All searches identified 7,612 references. After careful screening of the abstracts, 105 full papers were retrieved and included in the analysis because of their containing information relevant for the purposes of this review.

## Results

### Effect on OOP health spending

A large majority of the studies on OOP spending in the review that met our inclusion criteria focused on the catastrophic and impoverishing impacts of illness and suggest significant OOP healthcare expenses in LMICs due to health shocks (see Table 1).

**Table 1:** Effect of health shocks on household out-of-pocket health spending and impoverishment in low and middle income countries

**Study**	**Country**	**Data source**	**Out-of-pocket health expenditure (%)**	**Poverty incidence (%)**
Xu *et al*. 2003 [6]	59 countries	Household surveys 1991-2000	0-10.45 (40% of CTP)	-
Xu *et al*. 2007 [1]	89 countries	Household surveys 1990-2003	0-10.00 (40% of CTP)	-
Saksena *et al*. 2010 [12]	51 countries	World Health Survey 2003	0.62-29.96 (40% of CTP	-
Wagstaff & van Doorslaer, 2003 [5]	Vietnam	Living Standard Survey 1998	5.13 (40% of CTP)	3.40%^†^
			14.20 (10% of TE)	0.50%^‡^
Van Minh *et al*. 2012 [13]	Vietnam	Living Standard Survey 2010	4.60 (of TE)	2.50%^†^
			3.90 (40% of CTP)	
Garg & Karan, 2009 [14]	India	Consumer Expenditure Survey 1999-00	4.80 (of TE)	3.24%^‡^
			10.70 (of nFE)	
Joe & Mishra, 2009 [15]	India	Consumer Expenditure Survey 2004-05	6.10 (of TE)	4.40%^‡^
			12.00 (of nFE)	
Bonu *et al.* 2007 [16]	India	Consumer Expenditure Survey 2004-05	13.10 (10% of TE)	3.50%^‡^
			5.10 (40% of nFE)	
Gosh, 2011 [17]	India	Consumer Expenditure Survey 2004-05	5.51 (of TE)	4.40%^‡^
			15.37 (10% of TE)	
Arsenijevic *et al.* 2013 [18]	Serbia	Living Standard Measurement Survey 2007	5.00 (10% > up to	1.10%^†^
			20% of TE)	
Ico, RD. 2008 [19]	Philippines	Family Income and Expenditure Survey 2003	3.50 (10% of TE)	14.00%^†^
			3.80 (10% of CTP)	
Cavagnero *et al*. 2006 [20]	Argentina	National Survey on Household Expenditure & Conditions of Life Survey 1996-97	5.50 (40% of CTP)	1.70%^†^
Tomini & Packard, 2011 [21]	Albania	Living Standard Measurement Survey 2008	13.30 (of TE)	3.61%^†^
Mendola *et al.* 2007 [22]	5 Western Balkan countries	Living Standard Measurement Surveys 2000-2005	1.14- 26.32 (10% of TE)	0.05-2.80%^±^
van Doorslaer *et al*. 2006 [10]	11 Asian countries	Household surveys 1995- 2002	1.37-5.49 (of TE)	0.10-3.80%^||^
				0.30-3.60%^±^
Flores *et al.* 2008 [23]	India	National Sample Survey 1995–96 (Hospitalized cases)	29.20-34.15 (10% of TE)	7.24-7.91%^‡^
Su *et al.* 2006 [24]	Burkina Faso	Nouna Health District Household Survey 2000-01	8.66 (40% of nFE)	-
Gotsadze *et al*. 2009 [7]	Georgia	Health Care Utilization and Expenditure Survey 2007	11.70 (40% of CTP)	-
O’Donnell *et al.* 2005 [25]	6 Asian countries	Household surveys 1996-2002	2.98-15.57 (10% of TE)	-
van Doorslaer *et al.* 2007 [26]	14 Asian countries	Household surveys 1995-2002	2.01-15.57 (10% of TE)	-
			0.21-7.13 (40% of nFE)	

TE = total household expenditure.

CTP = ‘capacity to pay’.

nFE = non-food expenditure.

^‡^National poverty line.

^†^Subsistence poverty line.

^||^International poverty line of US$1.08 per day per person.

^±^International poverty line of US$2.15 per day per person.

In LMICs in Asia, OOP payments accounted for at least 30% of total healthcare expenditure in one set of multi-country studies [25,26]. Such OOP health expenditures have been associated with significant numbers of households incurring catastrophic levels of spending, and impoverishment: a study for 116 countries showed that globally each year healthcare payments pushed 100 million into poverty, and an estimated 150 million people exceeding a threshold (catastrophic) ratio of health spending to household’s ‘capacity to pay’ [1].

The studies summarized in Table 1 also show a fairly large variation in the impacts of illness on measures of catastrophic spending, ranging from 0% to 34% depending on the country, household survey and indicator used. Multi-country analyses by Xu *et al.*[1,6] estimated catastrophic spending in the range of 0% to 10% across countries using household income and expenditure surveys over the period 1991–2000, whereas Saksena *et al.*[12] estimated cross-country catastrophic spending variation from 0.6% to 30.0% of all households using World Health Survey for 2003. There were within-country differences as well. For example, in Vietnam, the proportion of households incurring catastrophic spending (defined as OOP equal to or greater than 40% of household’s ‘capacity to pay’) fell from 5.1% in 1998 to 3.9% in 2010, using data from Living Standards Measurement Survey [5,13]. However, aside from multi-country studies that use a similar threshold, cross-country comparisons are difficult because of the differing thresholds used for defining catastrophic spending: some studies used a threshold OOP share of total household expenditure; others used a threshold OOP share of household ‘capacity to pay’; and still others defined OOP thresholds as a share of non-food expenditure. In addition, the threshold share itself varied, ranging from 10% to 40%.

Estimates of the impact of illness on poverty among households also vary across countries, based on data from two multi-country studies: van Doorslaer *et al.*[10] for 11 Asian countries and Mendola *et al.*[22] for 5 East European countries, which range from 0.05% to 2.8%, using the World Bank $1/day and $2/day poverty line, respectively [10,22]. Individual country studies for Asia report even higher impoverishment impact of illness (3.2%-4.4% for India [14-17], 2.5%-3.4% for Vietnam [5,13], 14% for Philippines [19]). Tomini and Packard [21] also report 3.6% of households being impoverished by ill health in Albania (higher than the range of estimates reported in Mendola *et al*. [22] for 5 countries in East Europe that included Albania) [21,22]. With the exception of multi-country studies, however, comparisons across countries are confounded by the varying definitions of impoverishment used in the studies. For instance, individual country studies for Albania, Argentina, India, Serbia, Philippines and Vietnam, all used nationally determined poverty lines instead of the World Bank poverty lines used in Mendola *et al.*[22] and van Doorslaer *et al.*[10] that are similar across countries [5,10,18-22]. In addition, comparisons were made difficult due to the varying years for which estimates were calculated across countries. Concerns have also arisen about the existing methodology for measuring catastrophic spending and impoverishment in the literature. This is because one does not actually observe the ‘counterfactual’ – that is, household economic outcomes in absence of health shocks - for those who actually spent on health. One aspect of this concern is that if poor people are less likely to seek care, the poverty impact measure derived after deducting OOP healthcare payments may be downwardly biased. There is also an alternative scenario where the measure may be upwardly biased: if richer households are more likely to be insured and poorer households have to rely on borrowing or dissaving assets [22,27]. Using Indian household survey data Flores *et al*. [23] showed that ignoring the associated financing (coping) strategies of households in healthcare spending underestimated overall poverty impact significantly among households containing a hospitalized member.

To get around the problem of arbitrary thresholds, some studies (see Table 1) simply used the ratio of OOP to total spending (or non-food spending) although this information is available for a smaller set of countries (compared to catastrophic thresholds), primarily in Asia. These estimates ranged from 1.4% to 6.1% for a sample of 11 LMIC Asian countries (including a multi-country study by van Doorslaer *et al.*[10]) [10,13-15,17,25] and 13.3% for Albania [21]. Three studies reported OOP as a share of non-food expenditure (2 for India and one for Burkina Faso) [14,16,24]. Data for India also suggests that the share of OOP in total household spending rose over time from 1999–2000 to 2004–5 [14-17,23].

What are the major correlates of catastrophic spending and impoverishment, and generally higher OOP, among households? Our review of the literature also suggests that OOP spending has particularly serious effects on poor households, who tend to spend more on healthcare as share of their income compared to their richer counterparts [15,28-30]. Consequently, catastrophic health expenditure and impoverishment is disproportionately concentrated among the less well-off [1,5,6]. In a 5-country study of Eastern European countries, less well-off households already are disproportionately impoverished by OOP health payments despite the overall share of households experiencing catastrophic OOP healthcare spending declining [21,22]. The presence of dependents with members with disability and chronic illness, and elderly members in households are also linked to catastrophic expenses in some studies [24,28,31].

OOP health expenditures could also depend on the types of health care facilities and services used by patients when insurance is unavailable. Public facilities typically involve less OOP health spending than private facilities since they are subsidized, but the quality of services of public facilities in low-income settings is poor. OOP expenditures associated with a single hospital stay in a private facility for cancer or heart disease in India accounted for between 80-90% of annual per capita household income compared to 40%-50% of annual per capita income for care obtained at a public facility [32]. An analysis for Thailand also concluded that households using inpatient services from private hospitals are more likely to face impoverishment due to OOP spending [33]. Some multi-country studies have shown that OOP health spending is driven by health system characteristics such as the level of co-payments, prevalence of informal payments and the use of private providers [22]. However, the impact of insurance is less clear: in their systematic review, Acharya *et al.*[4] note that health insurance tends to lower OOP spending in some studies, but mostly the direction of impact is inconclusive.

#### ***Adult deaths***

Available studies on the impact of adult deaths suggest that OOP spending is generally higher among households with a recent death although not always statistically significant. In Ethiopia, households experiencing mortality among adults aged 15–54 years over a three-year period incurred a 7% higher share of health expenditure in total household spending compared to the households that did not experience mortality in that age-group [34]. In urban households of Vietnam, death of working age member in the 2 years preceding the survey led to households incurring a 27% increase in per capita medical spending in the last month which was not statistically significant differentiable from a hypothesis of no effect [35]. The recent death of any household member also increased per capita household OOP spending on healthcare by 27% in last one month in Laos [36], although again this was statistically not different from zero. In contrast, in Bangladesh the death of any household member in last two years decreased medical spending by 54% but medical spending for serious illness of any household member in last one year significantly increased by 62% [37]. Overall, the lack of statistical significance in many cases suggests that the results are best interpreted as inconclusive.

#### ***HIV***

Because HIV constitutes a major chunk of the global disease burden, it is of interest to assess its economic burden on households in LMICs. Although the number of studies we could access was limited, the weight of the evidence suggests that households with an HIV-positive member incur higher levels of OOP spending. In Coˆte d’Ivoire mean OOP health expenditures for the adults taking antiretroviral therapy were $24.3 per month, with 12.3% of households incurring catastrophic health expenditures based on the criterion of 40% of the ‘capacity to pay’ [38]. In South India median OOP medical and non-medical expenditures for treatment and services of HIV were US$ 122 in a reference period of six months [39] and in Malaysia, median OOP health expenditure for HIV-affected per patient per year were 14.7% of the patient’s median income [40]. In Nigeria, Mahal *et al.*[41] found per capita OOP expenses among households with HIV patients to be significantly higher than per capita OOP expenses of similar households but without an HIV patient as member.

#### ***Non-communicable conditions***

Recent studies in four South Asian countries (Bangladesh, India, Nepal and Sri Lanka) and Eastern Europe (Ukraine and Russia) show that households containing a member with NCD experienced significantly higher OOP health spending [42-44]. Two of the studies compared households containing a member with angina to a set of socioeconomically and demographically similar households but without a member with angina [43,44]. A third study found that the share OOP spending on healthcare in total household expenditure for households containing a member with heart disease was 16.5% higher relative to a set of socioeconomically and demographically similar households in India [45]. Mahal *et al*. [46] found that households containing a member with cancer experienced significantly higher OOP health expenditures per capita, relative to a set of matched controls. A study for Russia sought to correct for endogeneity to show that that households containing members with chronic diseases incur higher levels of OOP healthcare expenditure than those without [42].

### Effect on household labour supply and income

Table 2 summarizes key studies identified by our review that contain evidence of the impacts of health shocks on labour supply and income. These studies highlight the use of four main indicators of health shocks in the literature: adult death in the household, a measure of disability (e.g., indicator of activities of daily living (ADLs)), changes in self-reported health and specific disease indicators (e.g., heart disease).

**Table 2:** Effect of health shocks on household labour supply and income in low and middle income countries

**Study**	**Country**	**Data source**	**Statistical model**	**Measure of health shocks**	**Labour supply effect**	**Income effect**
Gertler & Gruber, 2002 [47]	Indonesia	Indonesian Resource Mobilization Study panel (1991, 1993)	Ordinary Least Square (OLS), Instrumental Variable (IV)	Change in index of limitations in household’s head ability to perform activities of daily living (ADLs). Index based on a formula using self-reported ability to perform basic and intermediate activities of daily living.	(-)7.60% in hours relative to baseline	(-)10% per capita of baseline earnings
Yamano & Jayne, 2004 [48]	Kenya	Rural household survey panel (19997, 2000)	Difference-in-difference (DID), OLS	Any adult death; Death of male household head		(-)35-40% off-farm income
						(-)79%*off-farm income
Beegle, 2005 [49]	Tanzania	Kagera Health and Development Survey 4 panels (1991-1994	Fixed effect regression & Probit model	Death of an adult household member (15–50 years) due to AIDS	(-)66-75%** men’s wage employment within 6 months	…….
Lindelow & Wagstaff, 2005 [50]	China	China Health and Nutrition Survey panel (1991, 1993, 1997, 2000)	Fixed effect regression	Worsening of self-assessed health (SAH) of household head by one rating on a 4 point scale (excellent, good, fair and poor) = small health shock; difference of 2–3 ratings = ‘large health shock’	(-)15%* labour market participation	(-)6.20%* total per capita income
						(-)10%* earned per capita income
Wagstaff, 2005 [51]	Vietnam	Vietnam Living Standard Survey panel (1993, 1998)	Fixed effect regression	Decline in log of average body mass index (BMI) among household members aged 18 plus between 1993 and 1998	…….	(-)59.90%** total per capita income
						(-)102.60%*** earned per capita income
Mete & Schultz, 2006 [52]	Taiwan	Surveys of Health and Living Status panel (1989, 1993, 1996)	Ordered probit model	Heart disease among elderly male;	(-)27.30%*** labour-force participation	…….
				Stroke among elderly male	(-)72.80%*** labour-force participation	
Wagstaff, 2007 [35]	Vietnam	Vietnam Living Standard Survey panel (1993, 1998)	Fixed effect regression	Death of working age member in urban areas in two or so years before the 1998 survey	…….	(-)26%*** total income
						(-)36.50%*** earned income
Bridges & Lawson, 2008 [53]	Uganda	Ugandan national household survey (2002–2003)	Heckman two-part model	Self-reported ill health (female); Self-reported ill health (male)	(-)6.20*** in paid employment	…….
					(-)3.90*** in paid employment	
Yamauchi *et al.* 2008 [54]	South Africa	KwaZulu-Natal Income Dynamics Study panel (1998, 2004)	Conditional fixed effect logit	Prime-age adult (20–44 years) mortality due to AIDS	(+)20%*** labour force participation (adolescents & female adults)	…….
Khan, 2010 [37]	Bangladesh (Dinajpur)	SHAHAR household survey 3 panels (2002–2003)	Fixed effect & random effect regression	Death of a household member in past 2 years; Serious illness of a household member that prevented from doing normal activities in past 1 year	(-)8.63 hours worked in the past week	(-)12.00% per capita earned income last month
					(-)2.61 hours worked in the past week	(-)8.65%* per capita earned income last month
Ghatak & Madheswaran, 2011 [55]	India	National Sample Survey (2004)	Tobit model	Not able to work due to ailment (illness)	…….	(-)21.60%** annual household income
Kadiyala *et al*. 2011 [34]	Ethiopia	Panel Ethiopian Rural Household Survey panel (1994–1997)	DID, Propensity Score Matching (PSM)	Prime age adult (15–54 years) mortality between 1994 and 1997	(+)dependency ratio = 0.32***	…….
Rocco *et al.* 2011 [56]	Egypt	Household Health Utilization and Expenditure Survey, 2002	Fixed effect regression & IV	Self-reported persistent health problem (disability, disease, injury or any other chronic disease) for at least 3 months during last 12 months	(-)26%*** being employed	…….
					(-)24*** hours per week	
Omar Mahmoud & Thiele, 2013 [57]	Zambia	Two-wave household panel (2001, 2004)	DID & PSM	Any prime age (12 years+) death between 1996 and 2001, and after 2001	…….	(-)4000-78000 (Zambian Kwacha) per adult-equivalent household income
Bales, 2013 [58]	Vietnam	Household Living Standards Survey panel (2004, 2006)	Fixed effect Poisson regression	Adult (15–60 years) member bedridden due to illness for 14 days or more in 12 months; Onset of disability (with respect to sight, hearing, memory and concentration, walking and climbing stairs, self-care and understanding and making oneself understood)	(-)7.70%** annual workdays	…….
					(-)11.90%* annual workdays	

Statistical significance at the level of 1%***, 5%** and 10%*; …….results not available.

#### ***Adult deaths***

Based primarily on small set of studies for African countries and one Asian country (see Table 2), it does appear that the effect of adult mortality is to lower labour supply in the households. However, this conclusion is clouded by the fact that the studies use varying outcome measures, ranging from work participation, labour force participation, work participation among specific demographic groups to the ratio of non-workers to workers in the household and varying reference periods. For instance, in Tanzania, men aged 20–50 years were 66%-75% less likely to participate in wage employment in the 6 months prior to death in households that experienced an adult death due to AIDS [49]. In Bangladesh, the death of a household member in the preceding two years lowered work participation of household members by an average of 8.6 hours in the last week [37]. Additionally in South Africa, the labour supply effects are in the opposite direction of increased labour force participation among a section of household members, namely adolescents and women [54]. Poor Indonesian households also increased labour supply by remaining members to compensate for income losses in the face of sickness and death [59].

Labour supply and work participation effects matter because they influence household income. For this reason, some studies sought to directly estimate the impact of adult mortality on household earnings and income. However, no clear conclusions can be reached. Analyses for Vietnam and Kenya suggest significant declines in income - ranging between 25% and 40% - for a household experiencing an adult mortality [35,48]. However one study from Bangladesh did not find statistically significant effect of adult mortality on household income [37]. A conclusion of no statistically significant effect of adult mortality on household income per capita was also reached by a study for rural Zambia [57].

#### ***Other health indicators (activities of daily living, self-assessed health, body mass index, any illness)***

Apart from adult deaths, Table 2 reports evidence of the impact of various generic health indicators (changes in level of disability, changes in self-assessed health, changes in body mass index (BMI) and any ailment) on labour supply and earnings. However, it is difficult to draw generalizable conclusions for a specific indicator given that disability and self-reported indicators as well as ailment (including severity of ailment) indicators used were very study-specific. In addition, indicators of self-reported health tend to be subjective across individuals, time and countries. Irrespective of the indicator used though, adverse health outcomes were associated with reduction in labour force participation and/or work-time. For instance, in Uganda, conditional on labour market participation, falling sick or getting injured over a 30-day reference period lowered the likelihood of being in the formal labour market by 4%-6% among adults [53]. A study of urban slums in Dhaka (Bangladesh) showed that more than 20% of adults out of 12 thousand interviewed took days off from work due to illness in 12 months preceding the survey [60]. In Vietnam, in households where an adult member (15–60 years) was bed-ridden for 2 weeks or more in the last year, annual work-days were lowered by 8% [58]. Larger health shocks are also likely to be associated with bigger income losses. While households in an Indonesian study were able to fully smooth income losses from minor illness (such as fever, respiratory congestion) and 71% of the income losses from moderate illness (defined as inability to perform intermediate ADLs: carry a heavy load for 20 meters; sweep the floor or yard; walk for 5 kilometres; take water from a well; and bend, kneel, or stoop), only 38% of the income loss from severe illness shock (defined as inability of performing basic ADLs: bathe yourself; feed yourself; clothe yourself; stand from sitting in a chair; go to the toilet; and rise from sitting on the floor) could be smoothed [47].

#### ***HIV***

Available studies, mostly for Africa, show that both labour supply and earnings decline in households affected by HIV. In South Africa, incomes of HIV-affected households are 35% to 50% lower than comparable unaffected households [61]. In Namibia, weight loss as proxy for an advanced state of AIDS is associated with a reduction in work time and earned income among the uninsured [62]. In Nigeria, HIV positive people experienced a decline in work participation (by 16 days) in a one year reference period and income losses due to sickness and caregiving amounted to about 40% of the combined healthcare costs and income losses compared to matched control households [41]. As noted previously, Yamane and Jayne [48] and Beegle [49], also found significant declines in labour force participation and earnings on account of adult deaths due to AIDS in Kenya and Tanzania, respectively [48,49].

#### ***Non-communicable diseases***

Studies of the impacts of NCDs on labour supply and income in households in LMICs are of recent origin, but they generally show household members work- and labour force participation declining as a consequence. Abegunde and Stanciole [42] showed that chronic non-communicable diseases were associated with reduced household income by 4.8%. Work participation among adult members of households containing an individual with CVD (cancer) was about 2%-3% lower relative to socioeconomically and demographically similar households but without cases of CVD (cancer) [45,46]. Heart disease and stroke caused significant declines in labour force participation by 27% and 73%, respectively among the elderly in Taiwan [52]. Outside of Asia - in Ukraine and in Egypt - two recent studies households containing a member with a chronic condition were likely to have lower rates of work participation [44,56]. It is not surprising that a member with a non-communicable condition will experience lower work participation, so that work and labour force participation by other household members is of obvious interest. However, little work exists on work force participation of non-sick household members in LMICs in the context of NCDs. Nor do we know much about the impact of health insurance on labour supply and employment in LMICs. The only recent study of which we are aware (for Thailand), showed that universal coverage discourages formal-sector employment among the Thai married men [8].

### Effect on household non-medical consumption

Household earnings losses and OOP healthcare expenditure resulting from health shocks can potentially lead to a decline in non-medical consumption, commonly used as a welfare indicator in economic analysis. Multiple studies have examined the effect of health shocks on household non-medical consumption in Asia, Africa and Latin America as indicated in Table 3. While the results vary in the magnitude of the impacts and are difficult to compare, either because they focused on specific sub-populations, or because varying methodologies (including indicators of health shocks) were used, non-medical consumption fell in nearly two-thirds of the studies we analyzed. Specifically non-medical consumption fell in 13 out of the 20 analyses of the impacts of health shocks reported in Table 3, rose in 6 cases and the results were unclear in one case.

**Table 3:** Effect of health shocks on household non-medical consumption in low and middle income countries

**Study**	**Country**	**Data source**	**Statistical model**	**Measure of health shocks**	**Non-medical consumption**	**Food consumption**	**Non-food consumption**
Dercon & Krishnan, 2000 [63]	Ethiopia	Ethiopian Rural Household Survey 3 panels (1994–1995)	Generalized method of moments	Females among poor Southern households are too weak to work in last 28 days	(-)1.70-2.30%*** body mass index (BMI) per month	…….	…….
Gertler & Gruber, 2002 [47]	Indonesia	Indonesian Resource Mobilization Study panel (1991, 1993)	Ordinary Least Square (OLS), Instrumental Variable (IV)	Change in index of limitations in household’s head ability to perform activities of daily living (ADLs). Index based on a formula using self-reported ability to perform basic and intermediate activities of daily living	(-)19.50% per capita	…….	…….
Asfaw & Braun, 2004 [64]	Ethiopia	Ethiopian Rural Household survey panel (1994, 1995)	Two-stage least square	Self-reported illness of household head within 4 weeks before the survey	…….	(-)1.80% last week	(-)33.59%*** last 4 months
Dercon *et al*. 2005 [65]	Ethiopia	Ethiopia Rural Household Survey panel (1999, 2004)	Panel regression	Death of head, spouse or another person; Illness of head, spouse or another person	(+)2.10% per capita	…….	…….
					(-)8.90%* per capita		
Wagstaff, 2005 [51]	Vietnam	Vietnam Living Standard Survey panel (1993, 1998)	Fixed effect regression	Negative changes in the log of average BMI among household members aged 18 plus between 19993 and 1998	…….	(-)17.30%* per capita	(-)16.90% per capita
De Weerdt & Dercon, 2006 [66]	Tanzania	Nyakatoke Household Survey 5 panels (February-December, 2000)	IV-regression	Medical expenditure and reduced labour supply due to due to illness	(-)7.30%* per adult	(-)4.80% per adult	(-)7.80% per adult
Beegle *et al.* 2008 [67]	Tanzania	Kagera Health and Development Survey panels (1991–2004)	Fixed effect regression, IV	Prime-aged (20–55 years) deaths due to AIDS (during 2000–2004)	(-)29.80%** annual per capita	…….	…….
Galiano & Vera-Hernández, 2008 [68]	Colombia	*Familias en Acci*o*n* household panel (2002, 2003, 2006)	Fixed effect regression	Any illness of adult male (aged 18–65 years) that does not let him perform ADLs in last 15 days	(+)US$9.65*** monthly	(+)US$4.46 * monthly	(+)US$3.87** monthly
Gertler *et al.* 2009 [69]	Indonesia	Indonesian Family Life Survey panel (1993, 1997)	Panel regression	Limitations in husband’s ADLs; Limitations in wife’s ADLs. Index based on a formula using self-reported ability to perform basic and intermediate activities of daily living.	(-)21.90% monthly per capita	…….	…….
					(-)17.20%% monthly per capita		
Khan, 2010 [37]	Bangladesh (Dinajpur)	SHAHAR household survey 3 panels (2002–2003)	Fixed effect regression	Death of any household member in past two years	…….	(-)15.30%* log per capita in last 3 days	(+)45.50%** log per capita in last month
Linnemayr, 2010 [70]	South Africa	Household survey 6 panels (2001–2003)	OLS	HIV non-affected household screened in last month; HIV affected households screened in last month	(+)27.70%*** monthly total	(+)25%***monthly total	(+)26.20%*** monthly total
					(+)2% monthly total	(+)2.20% monthly total	(+)2.20% monthly total
Wagstaff & Lindelow, 2010 [36]	Laos	Multi-shock cross-section survey (2008)	OLS	Death of any household member in last 12 months in the richest quintile	(-)67.90%*** annual per capita	(-)18.80% annual per capita	(-)107.20%*** annual per capita
Alem & Söderbom, 2012 [71]	Ethiopia	Household survey (2008–2009)	Probit regression	Self-reported illness of a family member; Death of a family member	(+)0.60% per adult equivalent	(-)2.70% per adult equivalent	……
					(-)11.10% per adult equivalent	(-)13.50% per adult equivalent	
Islam & Maitra, 2012 [72]	Bangladesh	Panel household survey (1998, 2000, 2005)	Fixed effect regression	Big expenditure/income loss due to illness; death of main family earner	…….	(+)0.02/100 Taka monthly	(+)1.05 per 1000 Taka yearly
						(+)0.31/100 Taka monthly	(+)1.64 per 1000 Taka yearly
Powell-Jackson & Hoque, 2012 [73]	Bangladesh	Household survey 2 panels (2007–2008)	OLS	Severe maternal complications (dystocia, haemorrhage, hypertensive disorders of pregnancy, septic shock or septicaemia, severe anaemia)	(-)5.30% monthly per capita	(-)7.50% monthly per capita	…….
Genoni, 2012 [74]	Indonesia	Indonesian Family Life Survey 2 panels (1997, 2000)	Fixed effect regression, IV	Deterioration in ability to walk 5 km; Deterioration in Intermediate ADLs (carrying a heavy load for 20 meters, walking for 5 kilometers, bowing or kneeling, sweeping the floor or yard, and drawing a pail of water from a well)	(+)1.60% monthly per capita	(-)4.20% monthly per capita	…….
					(+)1.20% monthly per capita	(-)3.60% monthly per capita	

Statistical significance at the level of 1%***, 5%** and 10%*; …….results not available.

There is also some evidence, from Ethiopia, that better-off households are able to protect their non-medical spending in response to health shocks [63]. At the other extreme, the very poor may be compelled to beg for survival in response to illness [60]. Gertler and Gruber [47] identified consumption effects of health shocks to be smaller where household heads are male, older and more educated and currently working. But Asfaw and Braun [64] and Dercon *et al.*[65] show that when head of the household is the person who is unhealthy, non-medical consumption declines sharply - ranging 15%-35% [64,65]. Similarly, the effect on illness on consumption depends on the type of health problem and type of health service used. For example, Wang *et al.*[75] found that in China, the adverse effects on consumption due to hospitalization were considerably greater than if a member suffered from a chronic disease, but was not hospitalized.

Evidence suggests that from LMIC households use a range of informal coping strategies to protect their non-medical consumption from health shocks (see Table 4). The use of current income and savings are often the immediate household response to financing OOP healthcare expenses following an illness [61]. But households also use relatively more of current income to finance moderate levels of OOP health expenditures when they are economically better-off or if the OOP spending is not excessive [23]. In Tamil Nadu (India), 70% of the better-off households used savings or income to finance OOP health spending compared to 55% among the poorest households [76].

**Table 4:** Coping strategies adopted by households in response to health shocks in low and middle income countries

**Study**	**Country**	**Data source**	**Statistical model**	**Measure of health shocks**	**Coping strategies**
Phung Duc & Waibe, 2009 [77]	Vietnam	Cross-sectional survey data, June-August 2007	Fixed effect regression	Idiosyncratic demographic shocks (death or illness of a household member) since 2002	11%-13%*** higher number of income sources used
Kruk *et al.* 2009 [30]	40 LMICs	World Health Survey, 2002-2003	Multiple logistic regression	Any health expenditure in last one year	***African households 87% and Southeast Asian households 61% more likely (compare to European households) to borrow or sell assets to finance health expenditure
Gertler *et al.* 2009 [69]	Indonesia	Indonesian Family Life Survey panel (1993, 1997)	Panel regression	Individual’s limitations in performing ADLs. Index based on a formula using self-reported ability to perform basic and intermediate activities of daily living.	***Smaller effects on consumption for households within 1 km of financial institution compared to within 10 km or more
Islam & Maitra, 2012 [72]	Bangladesh	Panel household survey (1998, 2000, 2005)	Fixed effect regression	Household incurred any big expenditure/income loss due to illness in past one years; Whether the main income earner died in the last one year	**Access to microcredit helps to insure consumption
Powell-Jackson & Hoque, 2012 [73]	Bangladesh	Household survey 2 panels (2007–2008)	Panel regression	Severe maternal complications (dystocia, haemorrhage, hypertensive disorders of pregnancy, septic shock or septicaemia, severe anaemia)	*** US$17 borrow per month, **US$4 asset sale and ***US$4.4 transfer per month compared to normal delivery to fully smooth consumption
Dercon & Krishnan, 2000 [63]	Ethiopia	Ethiopian Rural Household Survey 3 panels (1994–1995)	Generalized method of moments	Male or female household members are too weak to work in last 28 days	Household with more land are able to insure consumption
Asfaw & Braun, 2004 [64]	Ethiopia	Ethiopian Rural Household survey panel (1994, 1995)	Two-stage least square	Self-reported illness of household head within 4 weeks before the survey	Able to protect food consumption using own production and gifts
Park, 2006 [78]	Bangladesh	Matlab Health and Socioeconomic Survey, 1996	Two-stage least squares & Instrumental Variable	Income shocks out of death or illness of household members	**Relationship between neighbours and relatives helps in pooling risks to smooth food consumption
Sparrow *et al*. 2012 [79]	Indonesia	Socio-economic survey panel (2003, 2004)	Fixed effect regression	Household welfare affected during the last year by an event related to illness	15%*** used borrowing; 9%*** used selling assets;
					22%*** used family assistance; 9%*** reduced consumption
Abegunde & Stanciole, 2008 [42]	Russia	Life Standards Measurement Survey (8 rounds: 1997–2004)	Two-part Heckit model	Adults reporting chronic disease	7%*** increase in transfer income (gifts) per increase in household number of chronic diseases
Nguyen *et al.* 2012 [80]	Vietnam	Survey on 706 households (2008)	Multiple logistic regression	Hospitalization	Odds ratio = 18** (using loans);
					Odds ratio = 44* (reducing food consumption)
Raccanello *et al.* 2007 [81]	Mexico	Survey on 400 pawnshop users, 2005	Probit regression	Health expenditure due to persistence health shocks	(+) households used pawning to finance OOP health expenditure**
Modena and Gilbert, 2011 [82]	Indonesia	Family Life Survey, 1993	Poisson Multinomial Model	Demographic shocks (family deaths or illness)	(+) taking loans***;
					(+) selling assets***;
					(+) using family assistance***
Debebe *et al.*[83]	Ethiopia	Household survey, 2011	Probit regression	(self-reported illness, death or disability)	(+) 15%*** borrowed;
					(+) 17%*** used savings;
					(+) 17%*** sold assets;
Dhanaraj, 2014 [84]	India (Andhra Pradesh)	Young Lives survey panel (2006, 2009)	Multinomial logistic regression	Serious illness or death of father affected household economy negatively since the interviewer’s last visit	(+) 49%*** labour supply; (-) 93% *** consumption;
					(+) 53% borrowed or sold assets; (+) 54% received help
Alam & Mahal, 2014 [43]	4 South Asian countries	World Health Survey, 2002-2003	Propensity Score Matching (PSM)	Diagnosed or symptomatic angina	(+) 6-10%** households borrowed or sold assets to finance OOP health expenditure

Statistical significance at the level of 1%***, 5%** and 10%*.

Households that experience major health shocks, such as hospitalization or major illnesses (e.g., cancer, heart disease) tend to rely on borrowing or asset sales to finance their health expenditure [32,43,46,61,85-87]. Among poor households, informal borrowing, loans and sale of assets are frequently used to meet OOP healthcare spending due to common illness [23,30,73,80,82,83,88-90]. Sales of livestock were commonly used to finance healthcare in studies for Peru, Mexico and Bangladesh [72,91]. Income transfers from the broader community and the extended family are also protective of non-medical consumption as shown in a number of household level studies [42,87].

Conclusions about the impact of formal insurance mechanisms on non-medical consumption are not clear-cut. Most studies of the impact of formal health insurance in LMICs do not directly focus on the impact of insurance on non-medical consumption. The reviews by Acharya *et al.*[4] and Ekman [92] note, however, that health insurance is associated with lower OOP spending on healthcare in some studies, but that findings are unclear or even in the opposite direction in others [4,92]. Other than insurance, evidence from Bangladesh and Indonesia also shows that access to micro-credit institutions can help households to insure non-medical consumption against health shocks [69,72].

What emerges from studies in household inability (in many cases) to protect their non-medical consumption in response to health shocks, and the ability of better-off households to do this more effectively than their poorer counterparts are not upfront. Although households use a variety of strategies to ‘cope’ with the impacts of health shocks, these appear not be enough to protect their non-medical consumption. The inconclusive evidence on the impact of health insurance on OOP spending also suggests that such insurance may only provide partial protection against the impact of health shocks on non-medical consumption.

#### ***Adult deaths***

Available studies on the impact of adult deaths in LMICs point to unclear effects on non-medical consumption, with some studies pointing to a decline, others to no change and in at least one case to an increase in some components of household spending. In Laos, death of a household member in previous year in the richest quintile of households reduced overall household consumption by 68% and non-food consumption by 107% [36]. In Tanzania, prime-aged adult deaths lowered annual per capita household consumption by 30% [67]. In contrast, studies using data from Ethiopia and Bangladesh show no statistically significant effects of adult deaths on non-medical consumption [65,71,72]. A second study in Bangladesh found death of a household member lowered household per capita food consumption by 15% but increased per capita non-food consumption by 46% [37].

#### ***HIV***

Recent studies examining household level economic effect of communicable diseases on non-medical consumption are few and findings are mixed on the direction of the effects. In a study of South African households, there were no significant differences in household non-medical consumption between HIV-affected and unaffected households [70]. The influence of HIV on consumption may, however, have inter-generational consequences and some studies have tried to address this point. Specifically, two studies (also for South Africa) concludes that HIV-affected households in South Africa withdraw children from schools and spend less on food which might contribute to malnutrition [54,61].

#### ***Non-communicable diseases***

These too, are relatively limited, although a number of studies have recently become available for India. Karan *et al.*[45] show that households containing members with heart disease had lower per capita non-medical consumption (by 5 international dollars) over a 15-day reference period compared to set of socioeconomically and demographically similar households that did not contain a member with heart disease. Similar methods were also applied to compare households containing a member with cancer with a set of matched controls and the results showed that that households containing a member with cancer experienced lower non-medical consumption expenditure (by 66–85 Indian Rupees per household member) compared to matched control households for over a 15-day reference period [46]. However, a multi-country study in South Asia showed that non-medical consumption among households containing a member with angina did not statistically differ from socioeconomically and demographically similar households that did not contain a member with angina [43].

## Conclusions

The paper analyzed a large recent literature to explore the latest empirical findings for measuring the economic impacts of health shocks on households and the coping strategies. In the last decade, international literature in this area has tremendously grown covering more countries than ever from Asia, Africa, Latin America and some parts of Eastern Europe because of more availability of household level data. This has led existing reviews of the impacts of ill health in LMICs becoming both out of date and also geographically limited in their coverage.

Our main conclusions can roughly be summarized as follows. First, in the absence of formal health insurance households in LMICs tend to bear a high burden of OOP health expenditure, although there are considerable cross-country variations in household outcomes. This high OOP health payment for the household often stresses household’s ‘capacity to pay’ and pushes many households into poverty. Moreover, the economic burden of OOP health payments tends to be concentrated among the poor. In sum, protecting households from OOP health payments and subsequent catastrophic shocks continues to be a major health policy problem [1,6].

Second, difficulties in comparability across countries and studies notwithstanding, the overarching conclusion from the studies reviewed in this paper is that health shocks are likely to significantly reduce labour-days and labour income of the households in LMICs. Moreover, the adverse impacts are higher for health shocks of greater magnitude. The findings of the review about the impacts of health shocks on income losses are less clear with some studies showing a decline and others no effect at all.

A third, a conclusion is that the evidence generally rejects the assumption of full consumption insurance in the face of major health shocks. More generally, the more severe the illness, the less affected households were able to insure consumption. A few studies show that when households have access to credit at reasonable rates and they are fairly able to insure their consumption, such as when they have access to micro-credit [69,72]. Other household characteristics, such as socio-economic status including age, sex, education and employment status of the household members appears also to influence consumption smoothing of the households in the face of health shocks.

Fourth, the review identified a range of strategies households adopt in order to cope with the economic consequences of health shocks in LMICs. Although the adopted coping strategies are often context specific, the current review identifies using income, savings, borrowing, using loans or mortgages, and selling assets and livestock to meet OOP health spending of the households. Intra-household labour substitution, hiring external labour, and withdrawing children from schools are commonly used to compensate lost labour-days and income of the households. Also, access to informal credit from relatives, land ownership, and reducing non-medical consumption are used to protect the consumption (or food consumption, as appropriate) of the households.

The review did not explicitly look at the role of health insurance mechanisms in addressing household economic outcomes owing to the recent work of Acharya *et al.*[4]. However, as noted in that review, while there is some evidence of OOP reduction owing to insurance, there is cross-country variability and indeed evidence in some cases that OOP spending is unchanged or even rising in response to insurance. Very few studies have look at the implications of insurance for non-medical spending.

Finally, from a methodological perspective, the review noted that the comparability of the findings across countries and over time was hampered in many cases by the use of different indicators of the burden of OOP spending both within and across countries and different studies. A similar problem arose in the use of multiple indicators of labour supply used as outcomes, ranging from work participation to labour force participation and sometimes limited to specific age groups. In the absence of a counterfactual (household economic outcomes in the absence of illness) and inapplicability of randomized experiments to studying the impacts of disease means that the search for improved methods for identifying the impacts of illness is likely to continue.

Our review builds on previous reviews on the impacts of health shocks in multiple ways. The number of studies and countries covered in the group of LMICs is considerably larger than in previous work, much of it based on the pre-2000 literature. Moreover, it covers new topics not covered in previous work, including the emerging literature on impoverishment impacts of health and the implications of health shocks for non-medical consumption. The much larger pool of studies that we could choose them also meant that this review could limit itself to studies that met certain methodological thresholds, as in Acharya *et al.*[4]. Methodological differences notwithstanding, some of the findings of this review are similar to those in the previous review by McIntyre *et al.*[3] that OOP spending does frequently impoverish households, that adverse economic implications on households are influenced by the economic status of the household and severity of illness, and that households respond with a variety of coping strategies in response to health shocks similar to those we find. Nonetheless, we believe our review helps rest these conclusions on a much stronger empirical foundation than previously. As in previous reviews, there is always the risk of bias arising from the fact that studies reporting significant findings are more likely to be published.

Our review also helps shed some light on appropriate policy action and research avenues to pursue. Because the severity of health shocks, household economic status and health system characteristics matter for outcomes, policy makers will need to consider these factors in tailoring their social protection policies for specific sub-groups. Policy makers also need to consider non-health sector mechanisms, such as introducing of disability insurance, safety nets, or supporting existing informal mechanisms for the protection of households against losses in income and consumption from health shocks. Future research can answer the feasibility and effectiveness of such mechanisms in protecting low-income households from the overall economic consequences of health shocks. Other areas where research can be fruitfully directed, based on this review, include harmonization of indicators used for assessing health shocks and economic outcomes. Because these in turn reflect the exigencies of available data, there may be a need to better harmonize survey instruments as in the case of Living Standards Measurement Surveys and Demographic and Health Surveys. Additional work on the economic implications of NCDs for households is also needed given their current and future significance in the disease burden of LMICs. We also believe that there is scope for additional methodological work on the topics of measures of catastrophic health spending and impoverishment given the objections that multiple authors have raised about the existing methodology.

## Competing interests

The authors declare that they have no competing interests.

## Authors’ contributions

KA developed conceptual framework and search strategy with the guidance from AM. KA conducted the search, reviewed articles, prepared draft. AM reviewed the draft with substantive inputs on drafts of the manuscript. Both the authors read and approved the final manuscript.

## Authors’ information

KA is a doctoral student in Health Economics, and AM is his principal doctoral adviser. AM is the Finkel Chair in Global Health at Monash School of Public Health and Preventive Medicine, Monash University, Melbourne, Australia.

## Supplementary Material

Additional file 1 Definition of key variables. Click here for file
