# Supplementary material for: Economic impacts of health shocks on households in low and middle income countries: a review of the literature
Source: Global Health. 2014 Apr 3;10:21. doi: 10.1186/1744-8603-10-21 (PMC4108100; doi:10.1186/1744-8603-10-21)
Supplement: Additional file 1 — Definition of key variables. [file 1744-8603-10-21-S1.doc]

Additional file 1

| **Variable** | **Definition** |
| --- | --- |
| Health shocks | Health shocks have been defined in the literature in multiple ways: usually involving an event of death or disease or common illness with different degree of severity which impedes one’s ability to perform usual daily activities. Examples included in this review are:  - Inability of household member to perform basic activities of daily living (ADLs) (bathe yourself; feed yourself; clothe yourself; stand from sitting in a chair; go to the toilet; and rise from sitting on the floor) and intermediate ADLs (carry a heavy load for 20 meters; sweep the floor or yard; walk for 5 kilometres; take water from a well; and bend, kneel, or stoop). Usual method is to construct an index based on ability to perform ADLs and take a decline in the index as a health shock (Gertler and Gruber 2002)  - Worsening of self-assessed health (SAH) of household members (Lindelow and Wagstaff (2005) define a decline by one rating on a 4-point scale as “small health shock” and a 2-3 point rating decline as a “large health shock”)  -Change in Disability measured by an (composite) index of disability (CID) ≥ 4 on a scale of 0-7 range. |
| ‘Capacity to Pay’ (CTP) | ‘Capacity to Pay’ is a measure of disposable income once all basic survival needs are met. This can be constructed in multiple ways, depending on the definition of survival income (total expenditure minus subsistence needs, national poverty line, food expenditures, the World Bank’s international poverty line, etc.). |
| Out-of-pocket (OOP) health payments | Healthcare payments made by the household including all sources of health expenditures in a given reference period. |
| Catastrophic expenditure | When OOP health expenditure of a household or individual exceeds a certain threshold of total household income or ‘Capacity to Pay’. In practice, 5%, 10%, 15% and 20% thresholds have been used as threshold ratios of OOP to total income have been used for defining catastrophic spending; 10%, 20%, 30% and 40% threshold ratio of OOP to ‘capacity to pay’ have also been used to define catastrophic spending. |
| Impoverishment | A non-poor household is said to be impoverished by health spending if expenditure on non-medical items goes below the poverty line after OOP health expenditure is deducted from its total spending. |
| National poverty line | National poverty line is a country-specific poverty line derived based on cost of basic needs per person per day as defined by researchers/policymakers within each country. |
| Subsistence poverty line | Subsistence poverty line is defined as the minimum requirements for subsistence |
| Non-medical consumption expenditure | Total household expenditure minus medical expenditure. |
